# Supplementary material for: A new phased assembly of the Antarctic spiny plunderfish provides novel insights into the evolution of the notothenioid radiation
Source: Genome Biol. 2026 Oct 15;27:317. doi: 10.1186/s13059-026-04293-7 (PMC13670615; doi:10.1186/s13059-026-04293-7)
Supplement: Supplementary file 1 [file 13059_2026_4293_MOESM1_ESM.docx]

# **Supplementary figures**

**A new phased assembly of the Antarctic spiny plunderfish provides novel insights into the evolution of the notothenioid radiation.**

Jacopo Martelossi, Ksenia Krasheninnikova, Amy Denton, Jonathan M. D. Wood, Thomas Mathers, Richard Durbin, Nova Fong, David L. Bentley, Melody S. Clark, Iliana Bista

**Fig. S1:** Snail Plot of fHarAnt1.2 assembly. Top: Hap1, Bottom: Hap2.

**Fig. S2:** Counts of TTAGGG telomeric repeats over genomic windows of 10 kb across both haplotypes of the *H. antarcticus* genome.

**Fig. S3: A.** Genomic localization, and **B-C**. organization of nuclear rDNA genes.

**Fig. S4:** Structural comparison of Hap1 and Hap2 of fHantAnt1.2, and distribution of genomic features on Hap1, including genes, transposons, tandem and telomeric repeats. Black triangles: 18s, 28s and 5.8S genes, pink triangles: 5S.

**Fig. S5:** Proportion of heterozygous TE insertions identified on the *H. antarcticus* genome for different TE groups.

**Fig. S6:** Annotation of Zona Pellucida (ZP) genes for both haplotypes of *H. antarcticus*.

**Fig. S7: A.** Number and total amount of base pairs included in conserved elements (CEs) identified from the notothenioid whole-genome alignment and classified as shared by all notothenioids or only by cryonotothenioids. **B.** Example of a conserved non-coding element (CNE) upstream of *prdx5* acquired in the cryonotothenioid ancestor.

**Fig. S8:** Maximum likelihood phylogenetic tree of species included in selection analyses performed with aBSREL from the HyPhy package. Red colour indicates branches tested for positive selection in our focal (top) and in our control (bottom) analyses.

 **Fig. S9:** Annotation of the antifreeze gene locus of five notothenioid species. The genes have been annotated using GeMoMa and Klumpy. The verified gene annotation is shown in **Fig. 5D**. HarAnt: *Harpagifer antarcticus*, DisEle: *Dissostichus eleginoides*, NotRos: *Notothenia rossii*, PogAlb: *Pogonophryne albipinna*, PseGeo: *Pseudochaenichthys georgianus.*

**Fig. S10:** Annotated protein sequences of predicted chimeric *afgp/tlp* genes. The genes are shown on both haplotypes of the *H. antarcticus* assembly. Colours denote structural parts of the genes: Red letters: signal peptide, Yellow shaded: TLP mature protein, Red shaded: AFGP polyprotein, Grey shaded: XLF spacer.

**Fig. S11:** Read coverage (median) of 10X Genomics reads, and Nanopore reads over 500 bp windows across: **A**. the antifreeze gene locus plus flanking genomic regions, and **B**. the antifreeze locus only of Hap1 of *H. antarcticus*. **C**. Identity heatmap of the antifreeze locus obtained with StainedGlass.

**Fig. S12:** BLASTN dotplot between *afgp*14 and *afgp/tlp*2 genes of Hap1 in the *H. antarcticus* genome. The homologue region spanning exon-1 and part of intron-1 that was used for phylogenetic analyses is highlighted in red.

**Fig. S13:** Phylogenetic Maximum Likelihood (ML) trees including **A**. *afgp* + chimeric *afgp/tlp* genes, and **B**. *afgp*-only genes. Node labels represent UltraFast Bootstrap replicates.
